# Tea from the drinking to the synthesis of metal complexes and fabrication of PVA based polymer composites with controlled optical band gap

**DOI:** 10.1038/s41598-020-75138-x

**Published:** 2020-10-22

**Authors:** M. A. Brza, Shujahadeen B. Aziz, H. Anuar, Fathilah Ali, Elham M. A. Dannoun, Sewara J. Mohammed, Rebar T. Abdulwahid, Shakhawan Al-Zangana

**Affiliations:** 1 Department of Manufacturing and Materials Engineering, Faculty of Engineering, International Islamic University of Malaysia, Kuala Lumpur, Gombak, Malaysia; 2 Prof. Hameeds Advanced Polymeric Materials Research Lab, Department of Physics, College of Science, University of Sulaimani, Qlyasan Street, Sulaimani, Kurdistan Regional Government Iraq; 3 Department of Civil Engineering, College of Engineering, Komar University of Science and Technology, Sulaimani, 46001 Kurdistan Regional Government Iraq; 4 Department of Biotechnology Engineering, Faculty of Engineering, International Islamic University of Malaysia, 53100 Kuala Lumpur, Gombak, Malaysia; 5 General Science Department, Woman Campus, Prince Sultan University, P. O. Box 66833, Riyadh, 11586 Saudi Arabia; 6 Department of Chemistry, College of Science, University of Sulaimani, Qlyasan Street, Sulaimani, Kurdistan Regional Government Iraq; 7 Department of Physics, College of Education, University of Sulaimani, Old Campus, Sulaimani, 46001 Kurdistan Regional Government Iraq; 8 Department of Physics, College of Education, University of Garmian, Kalar, 46021 Kurdistan Regional Government Iraq

**Keywords:** Environmental sciences, Chemistry, Energy science and technology, Materials science

## Abstract

In the present study black tea extract (BTE) solution which is familiar for drinking was used to prepare cerium metal-complexes (Ce(III)-complex). The prepared Ce(III)-complex was characterized by Fourier transform infrared spectroscopy (FTIR), X-ray diffraction (XRD), and UV–Vis spectroscopy. The results indicate that BTE solution is a novel green coordination chemistry approach for the synthesis of metal complexes. The outcomes signify that coordination occurs between cerium cations and polyphenols. The synthesis of metal-complexes with superior absorption performance in the visible region is a challenge for optoelectronic device applications. The suspended Ce(III)-complex in distilled water was mixed with poly (vinyl alcohol) (PVA) polymer to fabricate PVA/ Ce(III)-complex composites with controlled optical properties. The PVA/Ce(III)-complexes composite films were characterized by FTIR, XRD, and UV–Vis spectroscopy. The XRD findings confirms the amorphous structure for the synthesized Ce(III)-complexes. The addition of Ce(III)-complex into the PVA host polymer led to the growth of polymer composites with controllable small optical band gaps. It is shown by the FTIR spectra of the composite films that the functional groups of the host PVA have a vigorous interaction with the Ce(III)-complex. The XRD deconvolution on PVA composites reveals the amorphous phase enlargement with increasing Ce(III)-complex concentration. It is indicated in the atomic force microscopy (AFM) that the surface roughness in the doped PVA films increases with the increase of the Ce(III)-complex. There is a decrease in absorption edge from 5.7 to 1.7 eV. It becomes possible to recognize the type of electron transition by studying both the Tauc's model and optical dielectric loss (ɛ_i_) parameter.

## Introduction

Even though consumption of tea was primarily for its central nerve stimulating and calming impacts, it has been associated with health improving effects for several years. It has been found that the health benefits associated with tea drinking include: anti-oxidant, anti-inflammation, cancer prevention, decreased occurrence of health disease, and so on^1^. Polyphenols in black tea consists of amino acid, alkaloids, theavins, catechins, isomers of theavins, caffeine and theanine. Dryan et al.^2^ performed a study recently, which showed that polyphenol compounds are widely prevalent in an aqueous mixture of black tea. The major components of tea are polyphenols, polyphenol conjugates, and polymerized phenolic structures. Furthermore, black, white, and green tea consists of a diverse mixture of conjugated flavonoids^3^. Controversies concerning advantages and risks of tea consumption still exist but the unlimited health-promoting advantages of tea surpass its few reported poisonous effects. The beneficial influences and risks related with tea drinking is well highlight in literature^4–6^. It was indicated in our earlier studies that a critical part is played by the extract solution of black and green tea in decreasing the optical band gap energy of polar polymers, like PVA and PMMA^7,8^.

It was indicated through the FTIR study that the extract tea solution is enriched with polyphenol, hydroxyl group, and carboxylic acid groups^8^. Hence, on the basis of the findings of the experimental studies, ample active functional groups and ligands are present in the tea extract solution, and these are critical for complex formation with heavy metal cations and/or polymers. A great risk and danger is posed by heavy metals, particularly when they are made part of the environment through pollution^9^.

Chemical precipitation, membrane technology, chemical coagulation, ion exchange, and electrochemical technology are amongst the different conventional techniques for removal and capturing of heavy metals. Nevertheless, these techniques are related with several drawbacks for example the necessity of large quantities of chemical additives, which create by-product sludge, large energy consumption, high cost, and small efficiency when the metals concentration are low^10^. Thus, development of clean, biocompatible, environmentally friendly techniques for capturing the cations of heavy metals and transfer them to metal complexes deserve merit.

In this study, the green technique has been employed to the synthesis of cerium metal complex (Ce(III)-complex) using extract solution of black tea. Extract solution of black tea leaves contains polyphenols to interact with the Ce^+3^ cations to synthesis Ce(III)-complex. Wang and An^11^ synthesized iron–polyphenols complex by extract solution of eucalyptus leaves leaves. The authors indicated that plant polyphenols cannot reduce iron ions to iron nanoparticles (NPs). The authors described that the plant polyphenols are capable to chelate iron cations to create iron–polyphenols complexes. Wang et al. in another study^12^ prepared iron–polyphenols complexes by sage (salvia officinalis) leaves extract solution. The authors determined that the polyphenols of the plant reduced gold and silver ions to gold and silver NPs, however only coordinated with iron cations to synthesis iron–polyphenols complexes.

In contrast to monovalent and divalent metal ions, cerium heavy metal ion (Ce^+3^) is trivalent ion which is not easily reduced to NPs and it is very active to make coordination with ligands and thus forming metal complexes rather than NPs. The Ce(III)-complex has good absorption behavior in the UV–Visible region, meaning that increase the optical absorption and light harvesting. Such absorption of a broader spectrum of solar radiation illustrates the utility of such material for applications. Thus, the formation of the Ce(III)-complex with enhanced absorption behavior in the visible region is of great importance from the industrial and device application viewpoints especially for organic solar cells, optoelectronics, and photonics. Moreover; the results of the present work can be recognized as a new approach in coordination chemistry which is related to metal complex synthesis. The Ce(III)-complex with this type of absorption behavior can be used in the preparation of polymer composites with controlled, and small optical band gap energies, as well as high performance optical properties. Additionally, Ce(III)-complex could be used to enhance the performance of the electrochemical energy storage devices. In our previous study it was displayed that the Zn(II)-complex insertion into the chitosan polymer electrolyte enhanced the performance of the electrochemical double-layer capacitor (EDLC) device^13^. Thus in addition to optical improvement; metal complexes are crucial to enhance the performance of the electrochemical devices.

In the current study, the Ce(III)-complexes are mixed with PVA polymer to prepare polymer composites with controlled optical properties. This technique can be considered as a novel green method to fabricate polymer composites with controllable optical band gaps. The analyses of the optical characteristics of polymers have been of much attention to researchers in the past few years because of their use in optical devices and their excellent properties of interference, reflection, anti-reflection and polarization^14,15^. It has been found in the latest studies that an important part is played by polymer composites that have low band gap energies and high absorptions for applications pertaining to photonics and optoelectronic devices^16,17^.

There are a variety of benefits of polymers, for example, simple processing, less expensive, flexibility, great strength, and excellent mechanical properties. PVA possesses large dielectric strength, excellent charge storage capacity, and its optical and electrical characteristics rely on the filler. PVA includes backbone chains of carbon with OH groups linked to the methane carbons. These OH groups are a source of hydrogen bonding and thus aid in the polymer blends creation^18^. PVA is a toxic free, greatly crystalline, soluble in water, biocompatible and biodegradable polymer^19^. A lot of interest has been given to polymers with varying optical properties in the past few years because of their role in the optical devices, sensors, and LEDs. It is possible to tune the optical properties of these materials by controlling the fillers and making the filler amounts optimal^20,21^.

Lately a lot of methods have been documented for the synthesis of metal complexes and preparation of polymer composites. It is recommended by the good optical properties in the current study that the green method is an exceptional method for synthesizing polymer composite films with small optical band gaps. It is suggested in the results that a valuable part may be played by the narrow band gap PVA having a good film forming in creating an equilibrium between cost and performance, and hence, solving issues like lifetime, flexibility and cost that cause a consistent decline in the use of conjugated polymers.

## Materials and methods

### Materials

Poly(vinyl alcohol) (PVA) powder material (average molecular weight = 85,000–124,000), cerium nitrate (Ce(NO_3_)_3_) [CAS Number 10294–41-4, Molecular Weight = 326.13 g/mol] was provided by Sigma-Aldrich (Kuala Lumpur, Malaysia). The black tea leaf was acquired from a local market.

### Preparation of Ce(III)-complex

The black tea leaves were used for extracting the natural colorant tea solution. For this purpose 40 g of black tea leaf was included in 250 mL of distilled water at 90 °C without being directly exposed to sunlight. After this, the solution was kept at ambient temperature to allow it to cool down. Whatman filter paper (Whatman 41, cat. No. 1441) that had a pore size of 20 µm was employed to eliminate the residues. In addition, 20 g of cerium nitrate Ce(NO_3_)_3_ was dissolved in 200 mL of distilled water. Ce(III)-complex was prepared by adding dissolved Ce(NO_3_)_3_ to the extract tea solution at a temperature of 60 °C. The solution was stirred for a total of 10 min. The colour of the tea solution changed from dark to green and precipitation took place with the development clouds at the lower end of the beaker, which suggests that metal-complex based material has been created. The solution that included Ce(III)-complexes were allowed to cool down till they reached room temperature, after which the centrifuge technique was used to separate the Ce(III)-complexes. The Ce(III)-complexes were washed with distilled water several times. After this, the Ce(III)-complexes were dispersed in 100 mL of distilled water.

### Fabrication of PVA based hybrid composites

The popular solution cast method was employed to fabricate the solid polymeric films. To prepare the PVA polymer solution, 40 mL of distilled water was added to 1 g of PVA powder and was stirred by a magnetic stirrer at 90 °C for 1 h. The PVA solution was then kept for 2 h to allow its temperature to decrease to room temperature.

From 15 to 45 mL of Ce(III)-complexes solution were added to the homogeneous PVA solutions separately in steps of 15-mL, after which the solutions were stirred consistently for 1 h. ORGCE0, ORGCE1, ORGCE2, and ORGCE3 were the codes used for the samples of PVA that included 0, 15 ml, 30 mL, and 45 mL of Ce(III)-complexes, respectively. Various Petri dishes (90 mm x 15 mm, Sigma-Aldrich, Kuala Lumpur, Malaysia) were used to cast the solutions, after which they were kept at room temperatures to dry so as to create the films. To allow the films to dry further, they were located in a desiccator with blue silica gel prior to optical characterization. The thickness of the pristine PVA and composite films are in the range between 0.0115 and 0.0185 cm.

### X-ray diffraction

X-ray diffractometer was performed to obtain the X-ray diffraction (XRD) at room temperature (Empyrean XRD-Panalytical), having an operating current of 45 mA and voltage of 40 kV. A beam of monochromatic was used to scan the samples, where the X-radiation had a wavelength of λ = 1.5406 A^°^, while the glancing angles ranged from 10^°^ ≤ 2θ ≤ 80^°^, having a step size of 0.05^°^.

### Fourier transform infrared (FTIR) spectroscopy

To examine the pristine PVA and composite films, FTIR spectrophotometer (Thermo Scientific, Nicolet iS10) was used in the wavenumber region 4000–400 cm^−1^ and having a resolution of 2 cm^−1^. The polymer thin films were cut into small circles with diameter of 2 cm and positioned at the sample holder of the FTIR spectrophotometer. The transmittance of the thin films of the pristine PVA and doped PVA were measured.

### Ultra violet–visible (UV–Vis) measurement

A Jasco V-570 UV–Vis-NIR spectrophotometer (Japan, Jasco SLM-468) was used to obtain the UV–vis absorption spectra of the samples within the absorbance mode. The absorbance of the pristine PVA and doped PVA were measured. Hence, the thickness of the polymeric films was greater than the thickness of the films were analyzed using the FTIR spectrophotometer. The absorbance data was used to examine absorption coefficient, extinction coefficient, optical band gap, and other optical parameters.

The definition of the absorption coefficient, which refers to the fractional decline in intensity per unit distance, is given below^22^:

1 $$ \alpha = \frac{ - I}{I} \times \frac{dI}{{dx}} = \frac{2.303}{d} \times A $$

Here, *I* refers to the intensity, *d* signifies sample thickness, and *A* is the absorption data.

Using the refractive index and extinction coefficient data, it becomes possible to experimentally determine the imaginary part of optical dielectric function (*ε*_*i*_) by carrying out the simple correlations given below^23,24^:

2 $$ \varepsilon_{i} = 2nk $$

Here, *n* refers to the refractive index and *k* signifies the extinction coefficient.

Determination of the optical band gap energy is indicative of the optical transitions in pristine PVA and doped PVA films. Tauc’s equation given below was applied to assess the optical band gap of the pristine PVA and related composite films^25^:

3 $$ \alpha = \frac{{A(h\upsilon - E_{g} )^{\beta } }}{h\upsilon } $$

In Eq. (3), the parameter dependent on the probability of interband transition is designated by *A*, the photon energy incident is referred by *hυ*, the band gap energy is designated by *E*_*g*_, and the β exponent specifies the type of electronic transitions.

### Atomic force microscopy

Atomic force microscopy (AFM) was used to to examine the doped PVA samples. The AFM was employed to examine the surface morphology of PVA:Ce(III)-complex composites. In the AFM, the surface of the doped PVA samples were scanned with a probe be composed of a miniature cantilever and a sharp tip. The tip fixed to a flexible cantilever was moved through the surface of the doped PVA samples to measure the surface morphology.

## Results and discussion

### FTIR analysis

**Figure 1:**
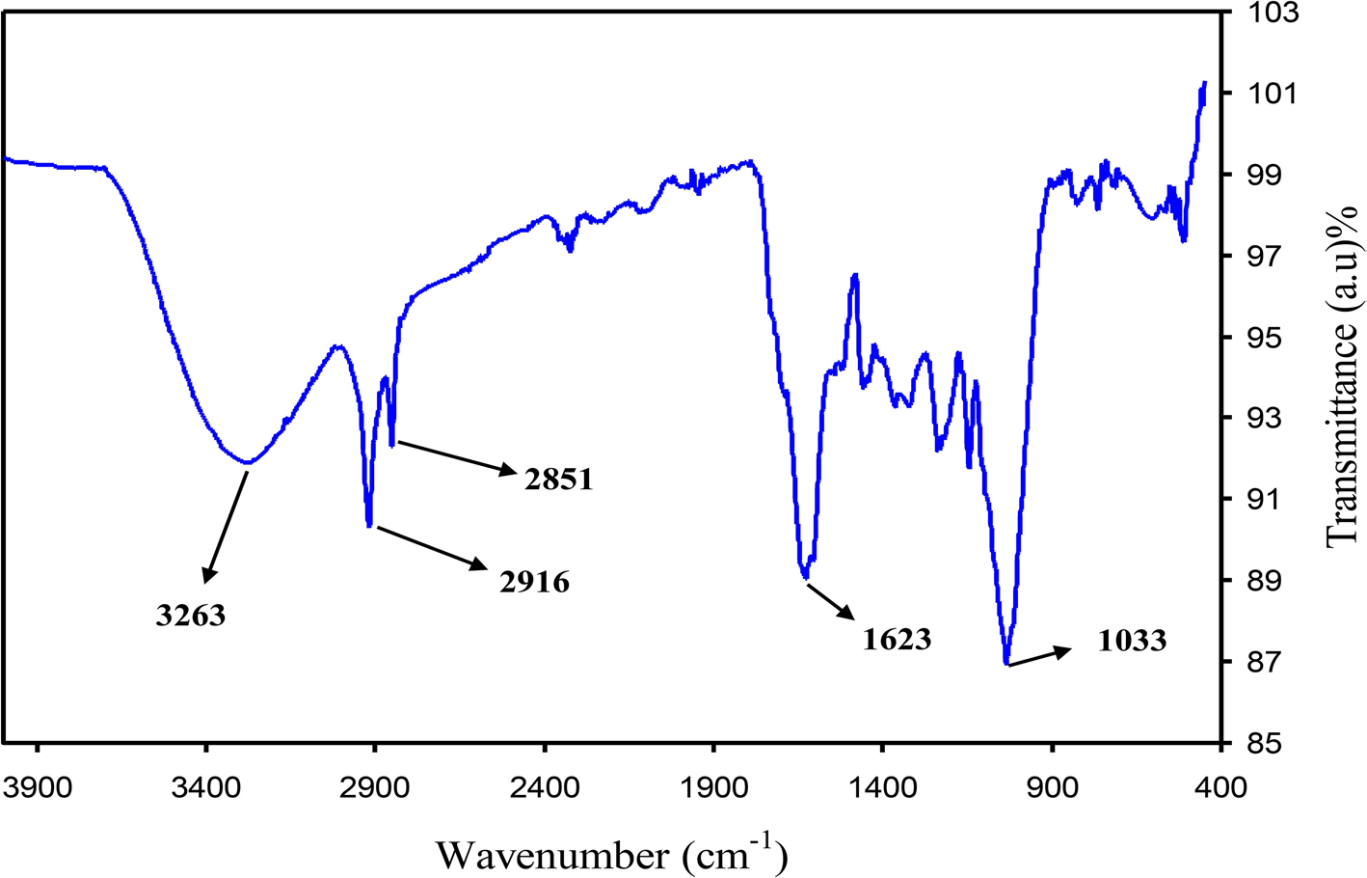
FTIR spectrum of black tea extract.

**Figure 2:**
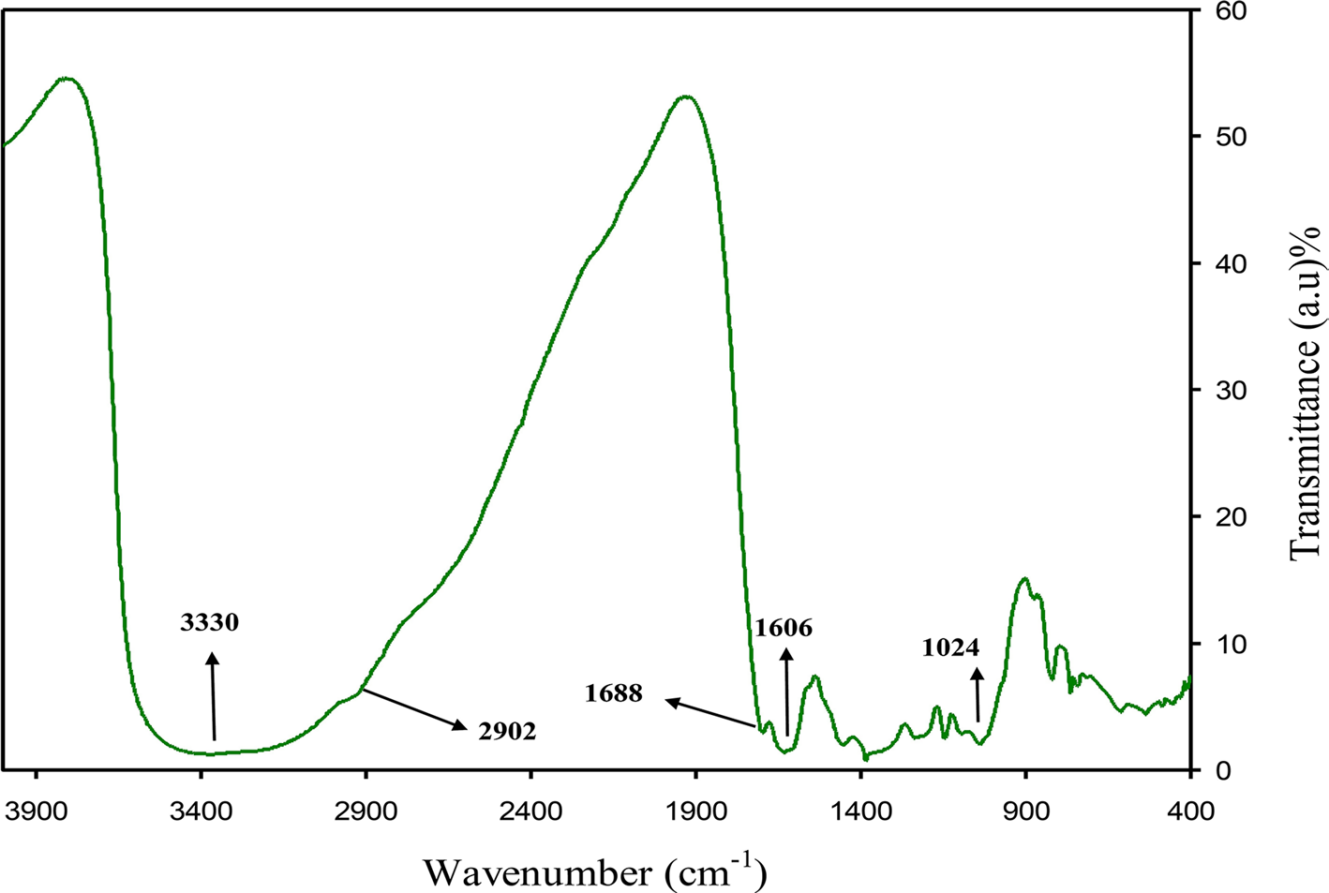
FTIR spectrum for Ce(III)-complex.

**Figure 3:**
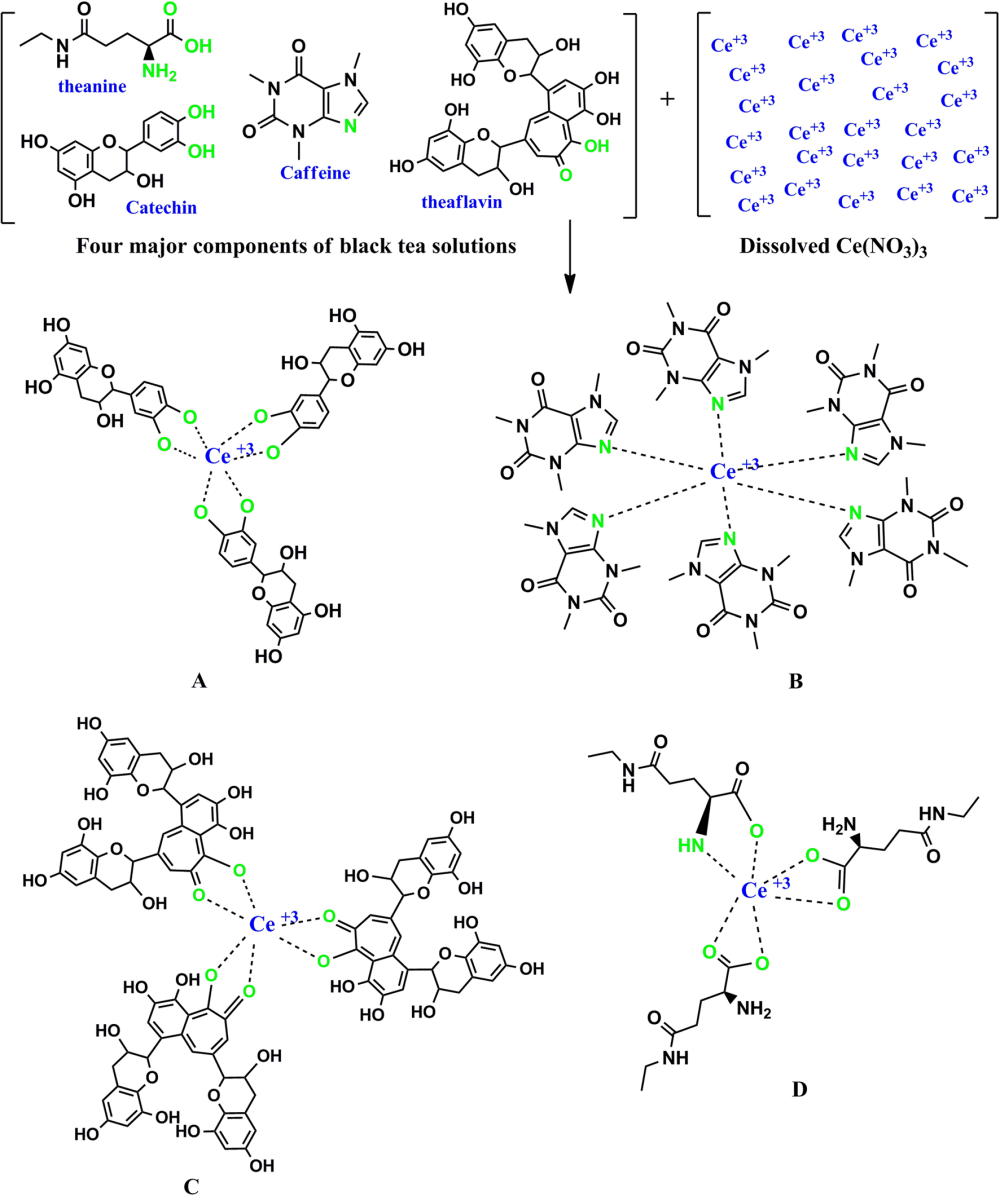
Theproposed structure for the Ce(III)-complex formation.

To characterize and recognize the molecules of extract solution of black tea leaf, Fourier-transform infrared (FTIR) spectroscopy was used. Several researchers used FTIR spectroscopy to examine different materials. The FTIR spectrum of the black tea (BT) extract solution is indicated in Fig. 1. A wide band was seen at 3401 cm^–1^, and it has been reported that it is linked to the O–H and N–H stretching modes of polyphenols^26,27^. It was stated by Saif et al.^28^ that the broad absorption band at 3310.7 cm^−1^ was linked to the hydroxyl (OH) functional groups in alcohols and phenolic compounds. It is also possible to allocate a strong band at 1628 cm^–1^ to the C=O bond stretching in polyphenols and C = C bond stretching in aromatic ring^27,29^. It was reported that the C–H stretch and O–H stretch in alkane and carboxylic acid surfaced at 2917 and 2848 cm^–1^, correspondingly^27^. A band has also emerged at 1029 cm^–1^ by the C–O stretching in amino acid^27,29^. Identical FTIR bands were also noted by Cai et al.^30^ in various types of tea for instance black, oolong, and green tea. It has been concluded in the previous studies that the FTIR bands of tea extract solution consisting of polyphenols emerged at 3388 cm^–1^, 1636 cm^–1^, and 1039 cm^–1^, and these are related to O–H/N–H, C=C, C–O–C stretches, correspondingly^27,29,31,32^. Hence, it is evident from the FTIR spectrum that the main functional groups in tea are polyphenols, carboxylic acid, and amino acid. Wu and Bird reported the colloidal suspensions of metal complexes and they believed that metal complexes are created due to the interactions of polyphenols, protein, and caffeine with the metal cations^33^. Hence, the idea of the current research is mainly to show that it is possible to effectively attribute cerium colloidal to Ce(III)-complexes using the FTIR technique. Figure 2 shows the FTIR spectrum of colloidal suspension of Ce(III)-complexes. The bands observed in FTIR spectrum of BT (see Fig. 1) are emerged again in IR spectrum of Ce(III)-complexes but with decrease and shifting in intensity (see Fig. 2). Recent investigations revealed that the modifications in the caffeine spectrum is noted in the range from 1700 to 400 cm^−1^ region as depicted in Fig. 2 corresponding of the binding and stretching vibrations of the pyrimidine, carbonyl, imidazole, and methyl fragments in the caffeine^34,35^. The absorption band intensity at 1033 cm^−1^ nearly vanished in the FTIR spectrum of Ce(III)-complexes and shifts to 1024 cm^−1^ (see Fig. 2). From the comparison of Figs. 1 and 2 it is noticeable that the peaks in the range between 1700 and 400 cm^−1^ nearly changes (see Fig. 2). The reason for this is that when coordination happens among Ce^+3^ cations, polyphenols, and caffeine, their vibration decreases owing to the cerium cations attachment and consequently their molecular weight increases. Recently, it was reported that theanine, catechin, caffeine, and theaflavin are the main components of black tea^36^. The proposed structure for metal cations with theaflavin is well reported by Coinceanainn et al.^37^. In their studies they established the formation of metal complexes thorough the FTIR, UV–vis and NMR studies. Thus, on the basis of the previous study and FTIR investigation of this research, the proposed complex formations of cerium ions (Ce^+3^) with the theanine, catechin, caffeine, and theaflavin of extract tea solution are indicated in Fig. 3. It is obvious from the proposed structure for the Ce(III)-complex formation that Ce^+3^ is capable to create complexation with catechin (see Fig. 3A), caffeine (see Fig. 3B), theaflavin (see Fig. 3C), and theanine (see Fig. 3D). Goodman et al.^38^, also indicated the complex creation between the metal cations and the extract black tea solution polyphenols via the examination of electron paramagnetic resonance (EPR) technique. In this work we displayed the metal complexes formation via the FTIR examination. For further confirmation we carried out XRD and UV–Vis investigation on metal complexes as can be seen in later section. It is well known that metal complexes possess good absorption from the visible to ultraviolet regions.

**Figure 4:**
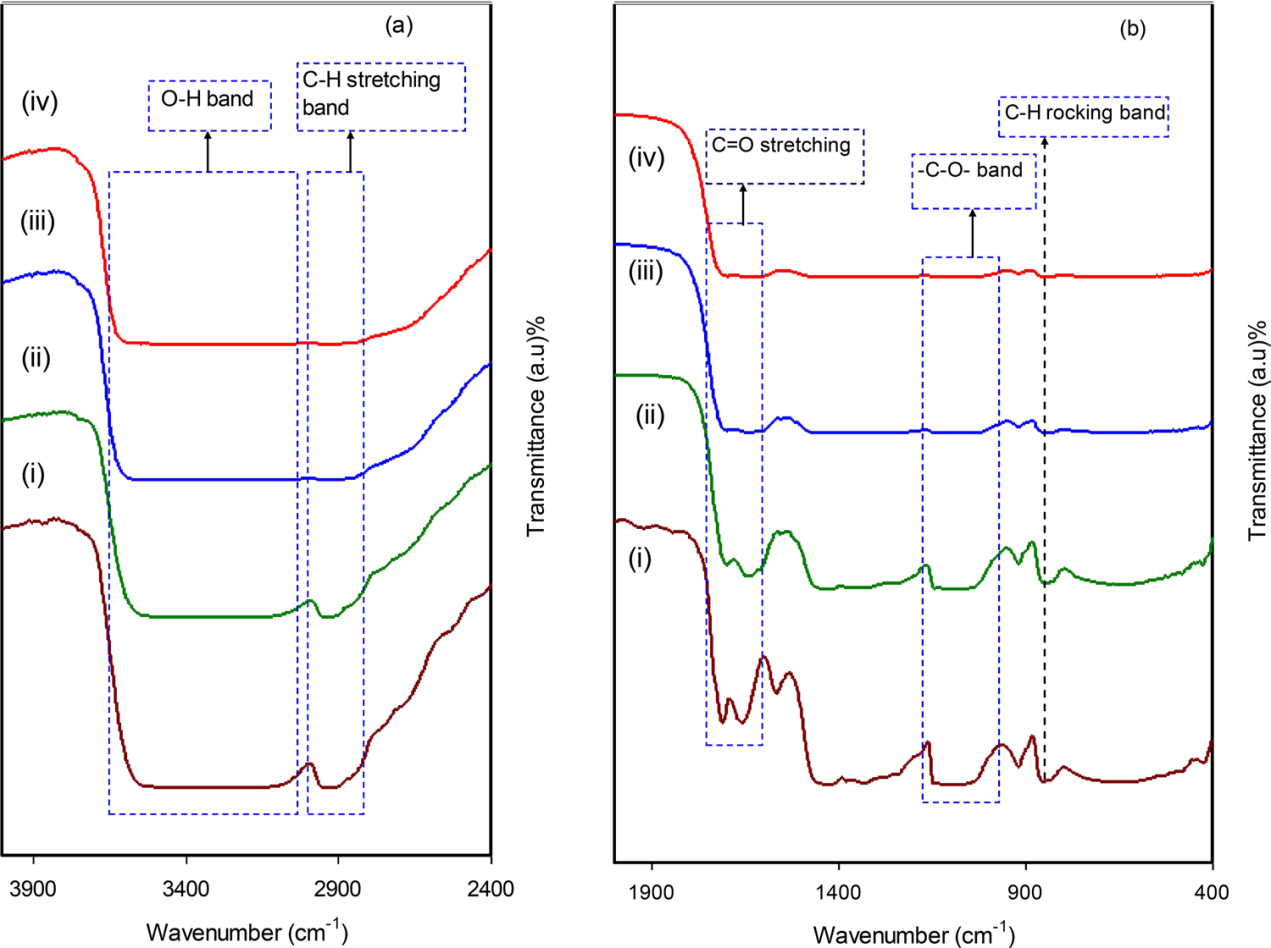
FTIR spectra of (i) ORGCE0 (pristine PVA), (ii) ORGCE1, (iii) ORGCE2, and (iv) ORGCE3 in the region (**a**) 400–1900 cm^−1^, and (**b**) 2400–4000 cm^−1^.

An important method to identify the interactions among ions or atoms in polymer composite or electrolyte systems is FTIR spectroscopy. The interactions that take place may cause modifications in the vibrational modes of the polymer composite^39^. Figure 4 displays the FTIR spectra of pristine PVA and PVA incorporated with various quantities of Ce(III)-complexes. The C-H rocking of pristine PVA has been allocated the absorption peak of 824 cm^−1^^40^. Interestingly, this peak changes and there is a decline in its intensity with an increase in the Ce(III)-complexes. This peak nearly diminished for 45 mL of Ce(III)-complexes. CH_2_ wagging and C-O plane bending are allocated the absorption peaks of pristine PVA at 1410 cm^−1^ and 1316 cm^−1^, respectively^41^. Within the doped samples, it is evident that the peak at 1316 cm^−1^ diminished. O–H stretching absorption of hydroxyl groups could be allocated a wide and strong absorption peak at 3340 cm^−1^ ^42^. It is the presence of strong intra and inter hydrogen bonds that caused this band to have high intensity^39^. There is a shift in the band in the doped samples, with its intensity falling to a large extent. C=O stretching vibration of acetate group is allocated the peak of pristine PVA at 1644 cm^−1^, and this is considered as the residual part of PVA^41^. There is a significant decline in the intensity of this peak when the concentration of Ce(III)-complexes is increased. The band relevant to C-H asymmetric stretching vibration takes place at 2908 cm^−1^^42^. For the doped samples, there is an evident shift and significant decline in this band. It can be seen in Fig. 4 that the peak at 1076 cm^−1^, which signifies the –C–O– stretch in pristine PVA^43^, shifts and experiences a decline in its intensity for the doped samples. Ce(III)-complexes link to the functional groups of the host PVA because of electrostatic or other types of interactions. This causes the vibrational intensity of the functional groups of the PVA polymer to decrease as greater molecular mass is obtained because of the binding of the metal complexes. Ultimately, its peak intensity experiences a shift as well as a decline^44^.

### XRD analysis

**Figure 5:**
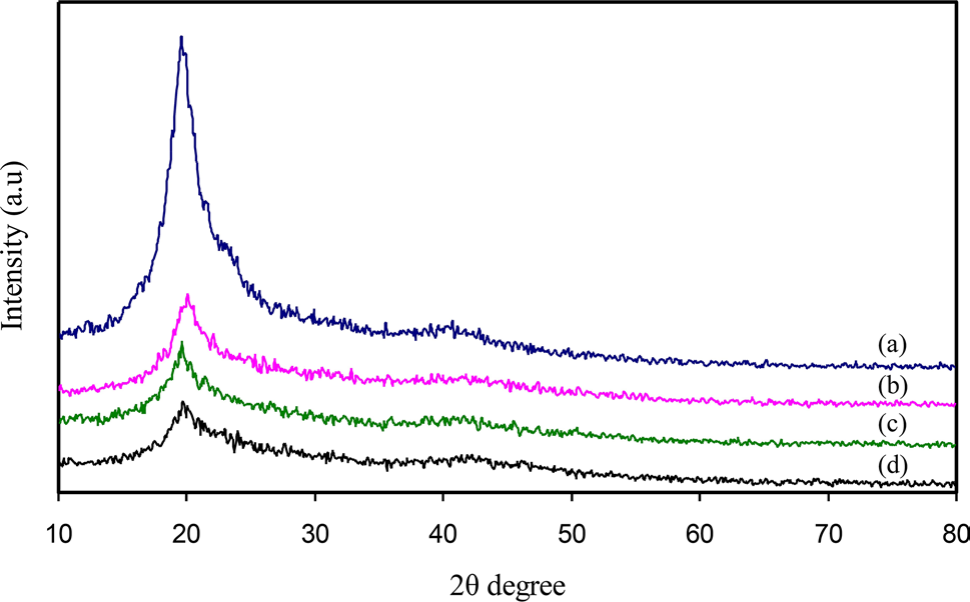
XRD pattern for (**a**) ORGCE0 (pristine PVA film), (**b**) ORGCE1, (**c**) ORGCE2, and (**d**) ORGCE3 composite films.

The XRD pattern of pristine PVA film is revealed in Fig. 5. It can be seen that two characteristic peaks are shown by pristine PVA film at 19.5° and 39.6^°^, and these are linked to the PVA semi-crystalline structure. The intermolecular and intramolecular hydrogen bonds support this semi-crystalline nature of PVA. These kinds of bonding might be created by the molecules in the individual monomer unit, or even in different monomer units^7^. There was a decline in the crystalline peaks intensity of pristine PVA (Fig. 5) when the Ce(III)-complexes was incorporated. There were no additional peaks within the XRD pattern of the PVA based polymer composites, suggesting that the Ce(III)-complexes within the polymer matrix are well distributed and complexed with the functional groups of the host polymer^45^. The hydrogen bonding disruption among hydroxyl and amino groups within PVA cause the widening and reducing in the relative intensity of the diffraction peaks of PVA when Ce(III)-complexes is added. Hence, the PVA composite samples experience amorphous improvement because complexion takes place between the added Ce(III)-complexes and the polymer matrix^46^. The standard put forward by Hodge et al.^47^ can be used to explain these results, which states that there is a relationship between the peak intensity height and the degree of crystallinity. It was noted by the authors that when the dopant was included, there was an improvement in the amorphous nature, which led to a decrease in the intensity of the XRD pattern^48,49^. Therefore, it is substantiated through the findings that complexion occurs between the PVA polymer and the synthesized Ce(III)-complexes. It is observed in Fig. 5 that when the amount of the Ce(III)–complex is increased, the peaks in ORGCE1 became smaller and less sharp (Fig. 5c,d). The insertion of Ce(III)–complex with 45 mL gives rise the smallest peaks, as shown in the XRD spectrum of ORGCE4 (Fig. 5d).

**Figure 6:**
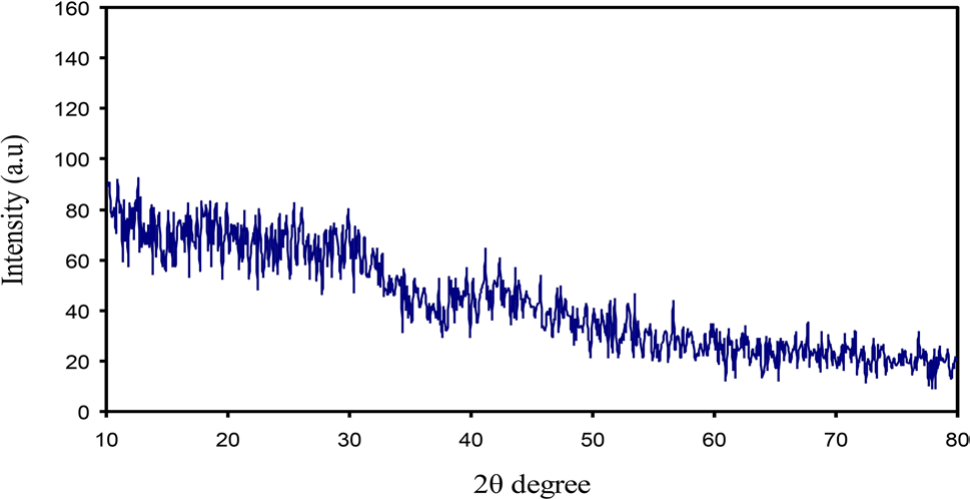
XRD pattern for synthesized Ce(III)-complex.

The XRD spectrum of Ce(III)-complex is displayed in Fig. 6, and designates that the synthesized Ce(III)-complex is amorphous as no crystalline peaks are observed through the 2θ degree ranges. The hump is only seen in the range between 2θ = 20° and 2θ = 30°. The XRD pattern achieved in this study is quite similar to that reported in the previous works^11,12,50^. Wang et al.^50^ fabricated iron-polyphenol complexes by plant extracts of rosemarinus officinalis, eucalyptus tereticornis, and melaleuca nesophila. The authors displayed that the XRD spectra for the three plants didn't show any distinct diffraction peaks, signifying that the iron-polyphenol complexes are amorphous.

### Morphological study

**Figure 7:**
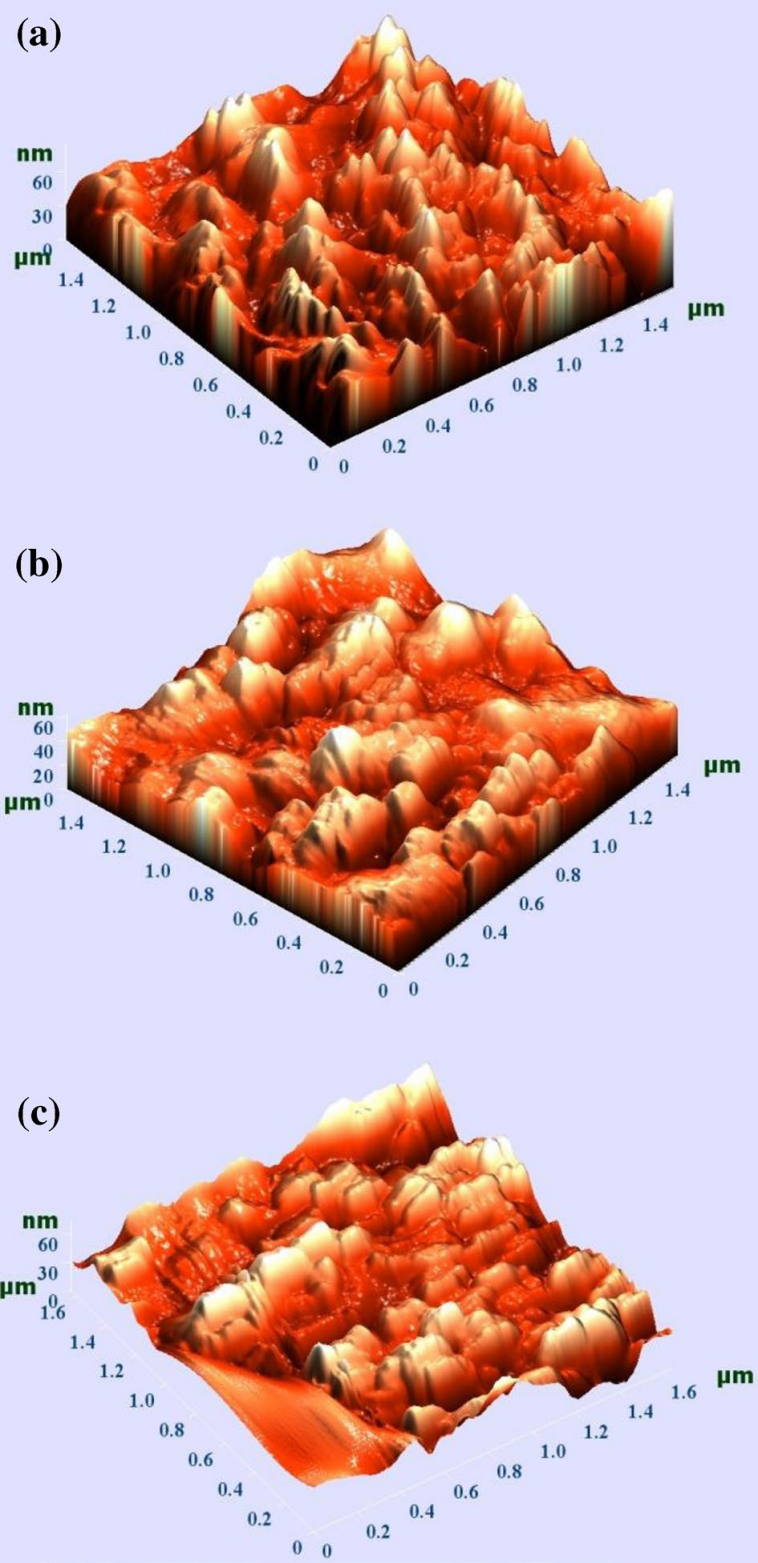
AFM 3D images for (**a**) ORGCE1, (**b**) ORGCE2, and (**c**) ORGCE3 composite samples.

Polymer macromolecules and polymer materials are distinguished by numerous analytical techniques that assist us to study their chain organization, chemical structure, mobility, nano-structure, morphology as well as various other properties. For instance, diffraction and microscopy techniques are extensively used for investigation of structural hierarchy in polymer films^51^. Atomic force microscopy (AFM) is employed in the laboratories of surface science to attain images with atomic resolutions of tenth part of nanometer. This kind of microscopy is efficiently useful in the polymers field to investigate the surface characteristics of the polymeric films^52^. As stated by Sousa et al.^53^, AFM investigation is vital to perceive the behavior of filler agglomerates in polymer composites. Furthermore, AFM is imperative to imaging periodic lattices of very drawn polymeric samples as well as single polymer crystals. The surface morphology of PVA:Ce(III)-complex composites are studied through the AFM as depicted in Fig. 7. AFM study is vital to examine the roughness of the polymer films. Obviously, with the increase of Ce(III)-complex, the surface roughness increases and the images showed phase separated heterogeneous morphology at higher concentration of Ce(III)-complex. This confirms the results obtained from the XRD measurements. Earlier study established that AFM produces a means for well comprehending the structural organization. Especially, AFM data can be used for the reasonable understanding of the results of diffraction methods^51^.

Sreekala and Eger used AFM^54^ to investigate the surface of the reactive epoxy resin/nanosilica (SiO_2_) using the so-gel process. Atomic force micrographs, captured from the fracture surface of the nanocomposites, showed the nanoparticles distribution. A uniform dispersion and good distribution of the nanoparticles were seen at all SiO_2_ concentrations. The authors indicated that at a volume content of minimum up to 14%, the nanoparticles improved mechanical properties. However, the nanoparticles had no substantial impact on the nanocomposites optical properties^54^.

### Absorption and absorption edge study

**Figure 8:**
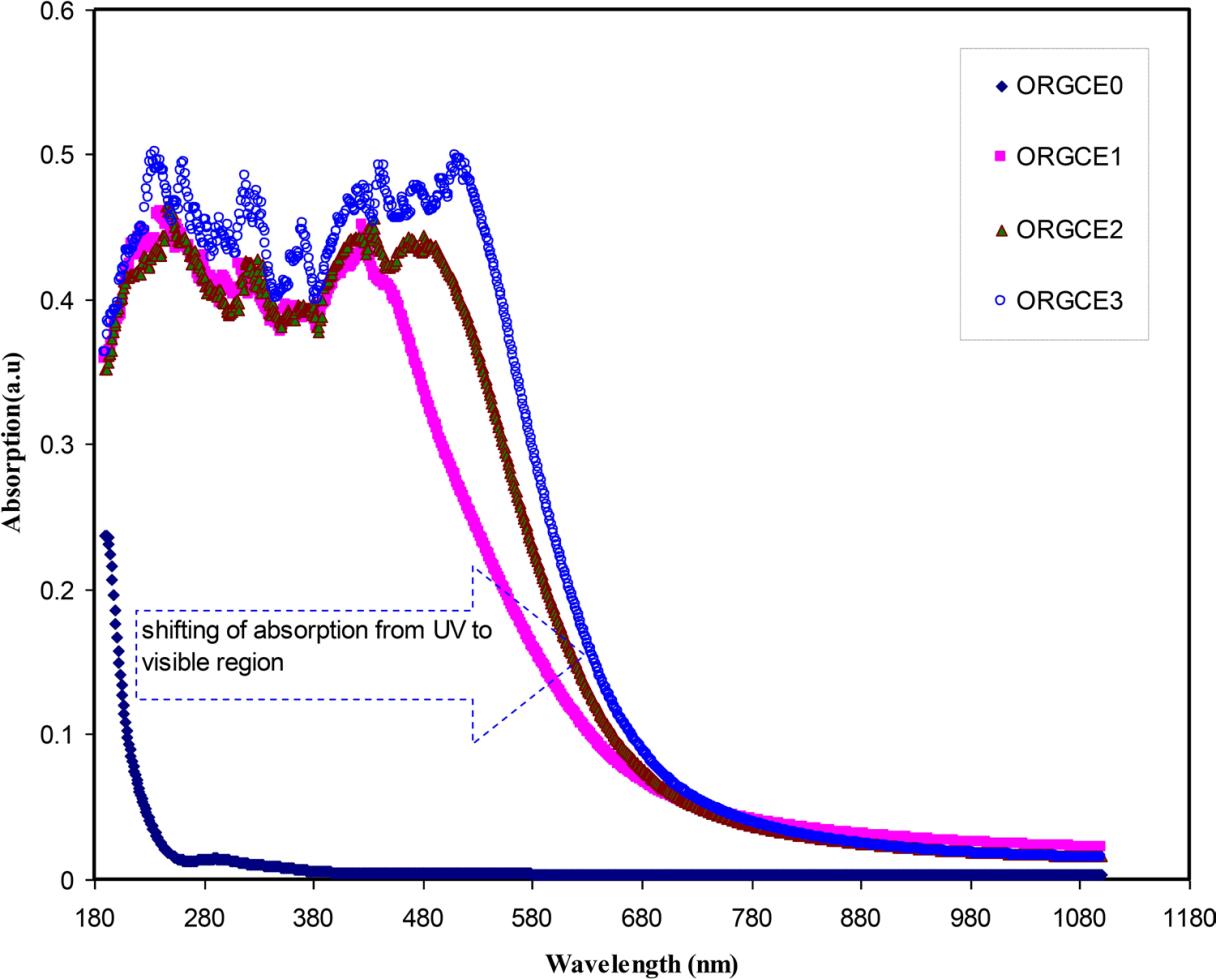
Absorption spectra of pristine PVA (ORGCE0) and PVA composite films.

The absorption spectra for pristine PVA and PVA composite films can be seen in Fig. 8. It is evident that absorption does not take place in the visible region of the absorption spectrum of pristine PVA, and hence, it is nearly transparent over these wavelengths. This is linked to the insulating characteristics of pristine PVA. There is clear evidence to suggest that when the energy of the incident photon is less than the energy difference between the two electronic levels, the photons absorption does not take place and the materials remain transparent to this photon. Absorption occurs when the photons energy is greater, and a transition is created by the valence electrons between the two electronic energy states^55^. It is believed that the shift and increase in absorption within the doped samples may be linked to the extensive absorption of Ce(III)-complexes.

**Figure 9:**
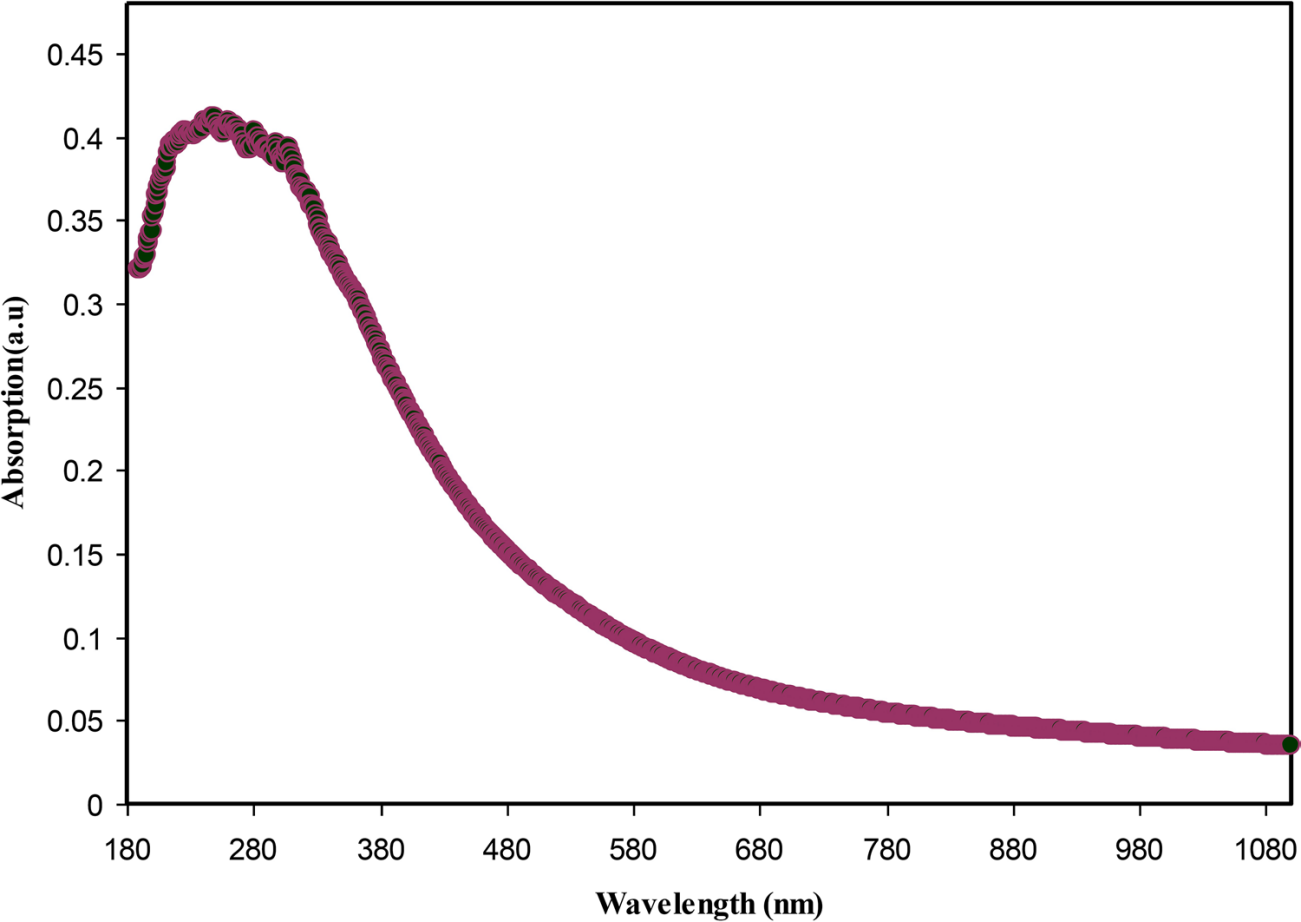
Absorption spectra for the synthesized cerium metal complexes.

Figure 9 shows the absorption spectrum of colloidal suspension of Ce(III)-complexes. It should be noted that the entire visible range is encompassed by the absorption, commencing from the visible range to the UV range. It is only the semiconductors and metal complexes that exhibit these kinds of absorption spectra^56–58^. Hence, it may be deduced that the natural dyes obtained from black tea are valuable environmentally friendly dyes used to capture the heavy metal ions and synthesize metal complexes. It has been shown in the latest studies that natural dyes are given a lot preference as they are known to be environmentally friendly. In addition, they also have other properties, for example, deodorizing, less toxic, anti-bacterial, anti-allergic and anti-cancerous^59,60^.

The peaks at 200–300 nm in the extract solution can be related to polyphenols. The absorbance of the wavelength in the range between 200 and 350 nm is linked with the n–π^*^ electronic transition of catechins and methylxanthines, which composed of theobromine, theophylline, and caffeine. The band absorbance at around 278 nm is related with the C = O chromophore of caffeine^61–63^. Metal nanoparticles must exhibit a surface plasmon resonance (SPR) peak in the region of UV − visible^64^. In the spectrum in Fig. 9, no SPR peak of Ce(III)-complex has been seen; meaning that synthesized Ce(III)-complex have no metal properties on the particle surfaces because of the capping of polyphenols. It was indicated in the previous study, chitosan-based polymer electrolytes display SPR peak, which created by copper nanoparticles in the range from 500 to 800 nm^65^.

The interband absorption process represents the process of transitioning electrons between the bands of a solid. A valuable technique for evaluating the band structure and energy gap of crystalline as well as non-crystalline materials is the optical absorption, specifically the absorption edge research^66^. It is evident that attenuation or losses are faced by a light wave with distance when it passes through a material. The absorption coefficient is given by Eq. (1).

**Figure 10:**
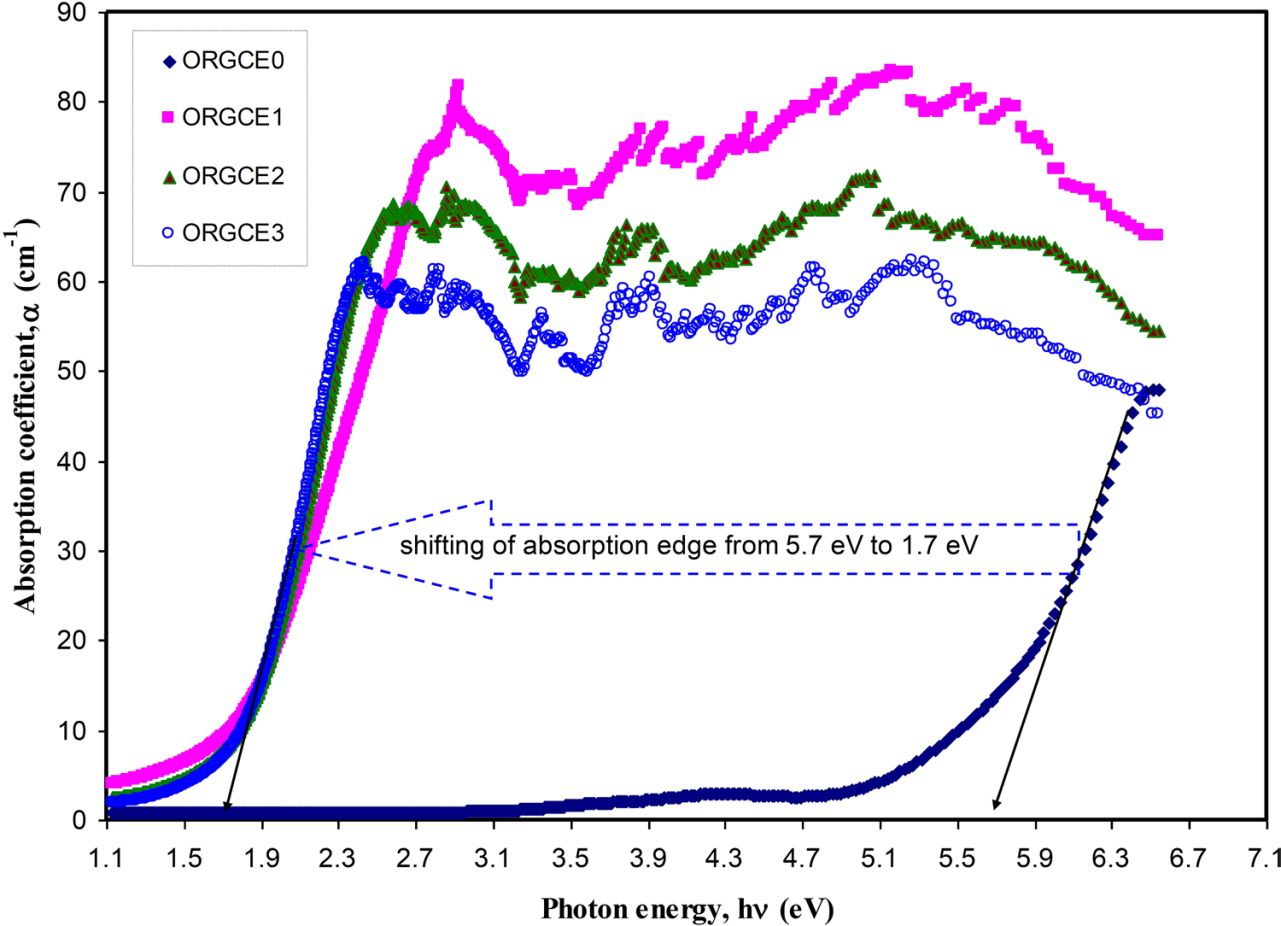
Absorption coefficient against photon energy for pristine PVA (ORGCE0) and PVA composites.

The absorption edge ( *E*_*d*_) is created when optical transitions start taking place over the fundamental band gap of a material^67^. Therefore, a new research domain in optical materials is created when the PVA is modified to narrow band gap polymer composite by incorporating green synthesized Ce(III)-complex. Subsequently, a novel technique for polymer composite fabrication by employing the green methods is established. The wide *E*_*d*_ shifting to lower photon energy is indicated in Fig. 10. There was an evident decrease in the value of *E*_*d*_, falling from 5.7 to 1.7 eV for the samples injected with 45 mL of colloidal suspension of Ce(III)-complex (Fig. 10). The absorption coefficient values obtained in the current study are quite close to the values found for the doped polyacetylene (trans-(CH) x), while being in close proximity of those obtained for polypyrrole (PPy)^68^. This has a direct link with the creation of charge transfer complexes within the composite samples^7,69,70^. Duvenhageet al.^71^ doped PMMA with Alq3 for being used in optoelectronics.

### Band gap study

Complex dielectric function linked to other definitive optical properties (reflectivity, refractive index, and absorption coefficient) can be used ideally to explain the optical properties of solids using simple equations^72^. It was determined in previous studies^69,70^ that optical dielectric loss (ɛ_i_) parameter using Eq. (2) and Tauc’s model using Eq. (3) could be employed to measure the optical band gap energy and specify the nature of electronic transitions, correspondingly. This is linked to the fact that there is hardly any relationship between optical dielectric function and materials band structure. Concurrently, the studies of optical dielectric function carried out by UV–vis spectroscopy are also quite valuable in anticipating the band structure of the materials on the whole^23,24,40^. An analysis of complex dielectric function (ε) can provide an improved understanding of the optical properties of a solid. The linear response of materials to the electromagnetic radiation is presented by this function^23,24^. To explain the structure–property relationship more accurately, quantum mechanics needs to be considered thoroughly, that is, examining the real and imaginary parts of a complex dielectric function $\varepsilon (\omega ) = \varepsilon_{r} (\omega ) + i\varepsilon_{i} (\omega )$ that is linked to other quantifiable optical quantities^73^. In terms of quantum models, there is a powerful relationship between the ɛ_i_ parameter and the analysis of optical band gaps. The ɛ_i_ represents the optical absorption within the material, and this has a strong relationship with the valence band (VB) and conduction band (CB), which is shown by^23^:

4 $$ \varepsilon_{i} (\omega ) = \frac{{2\pi \,e^{2} }}{{\Omega \varepsilon_{0} }}\sum\limits_{K,V,C} {\left| {\left\langle {\Psi_{K}^{C} \left| {\vec{u}.\vec{r}} \right|\Psi_{K}^{V} } \right\rangle } \right|}^{2} \delta (E_{K}^{C} - E_{K}^{V} - \hbar \omega ) $$

Here, ω refers to the incident photon frequency, Ω refers to the crystal volume, e signifies the electron charge, ε_o_ refers to the free space permittivity, $\vec{r}$ signifies the position vector, $\vec{u}$ refers to a vector described as the incident electromagnetic wave polarization, and $\Psi_{K}^{C}$ and $\Psi_{K}^{V}$ denote the CB wave function and VB wave function at *k*, respectively. On the basis of the theoretical models, a complex function of frequency is used to define the ɛ_i_ parameter, which needs an extensive computational effort to be computed^74–77^. Hence, extensive numerical techniques and durations are needed by the quantum models.

**Figure 11:**
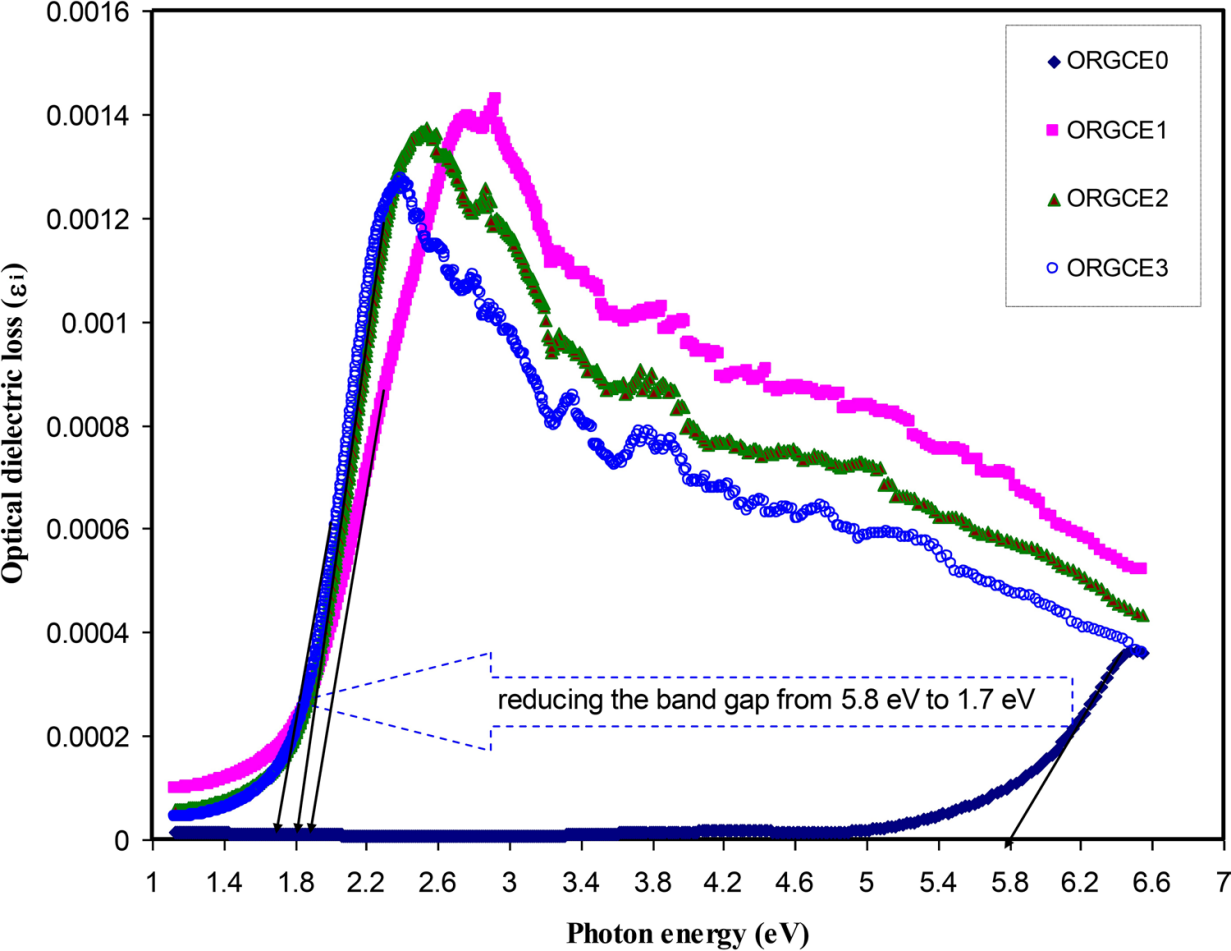
Optical dielectric loss spectra versus photon energy for pristine PVA (ORGCE0) and PVA composite films.

Experimentally the ɛ_i_ parameter is measured by Eq. (2). It was deduced in the previous studies that the peaks that emerged in the spectra of the ɛ_i_ have a direct correlation with the interband transitions^72,77–79^. Hence, it is possible to attain the real energy gap using the intersection of linear parts of ɛ_i_ spectra on the horizontal axis of photon energy (refer to Fig. 11). The ɛ_i_ parameter essentially signifies the transition of electrons from the filled states to the unfilled states^23,80^. It was affirmed in the previous studies that optical transition process could be comprehended extensively by assessing the ɛ_i_ parameter^77^. Interband transition can take place, where a photon causes an electron to jump from a filled level in the VB to an unfilled level in the CB. The photon is absorbed in this process; an excited electronic level is created, leaving behind a hole. This process is inherently quantum mechanical^81^.

**Figure 12:**
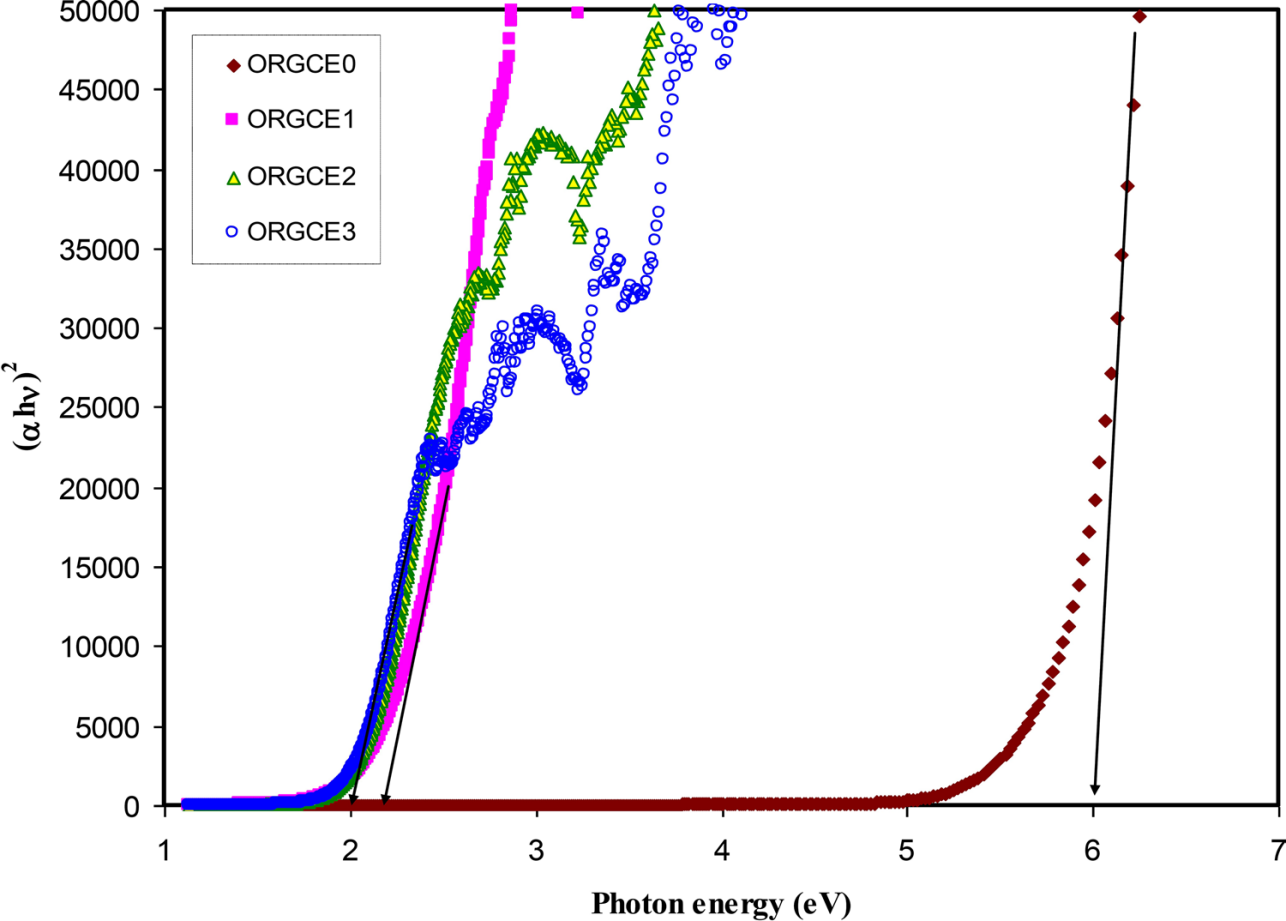
Plot of *(αhυ)*^*2*^ vs photon energy for pristine PVA (ORGCE0) and composite films.

**Figure 13:**
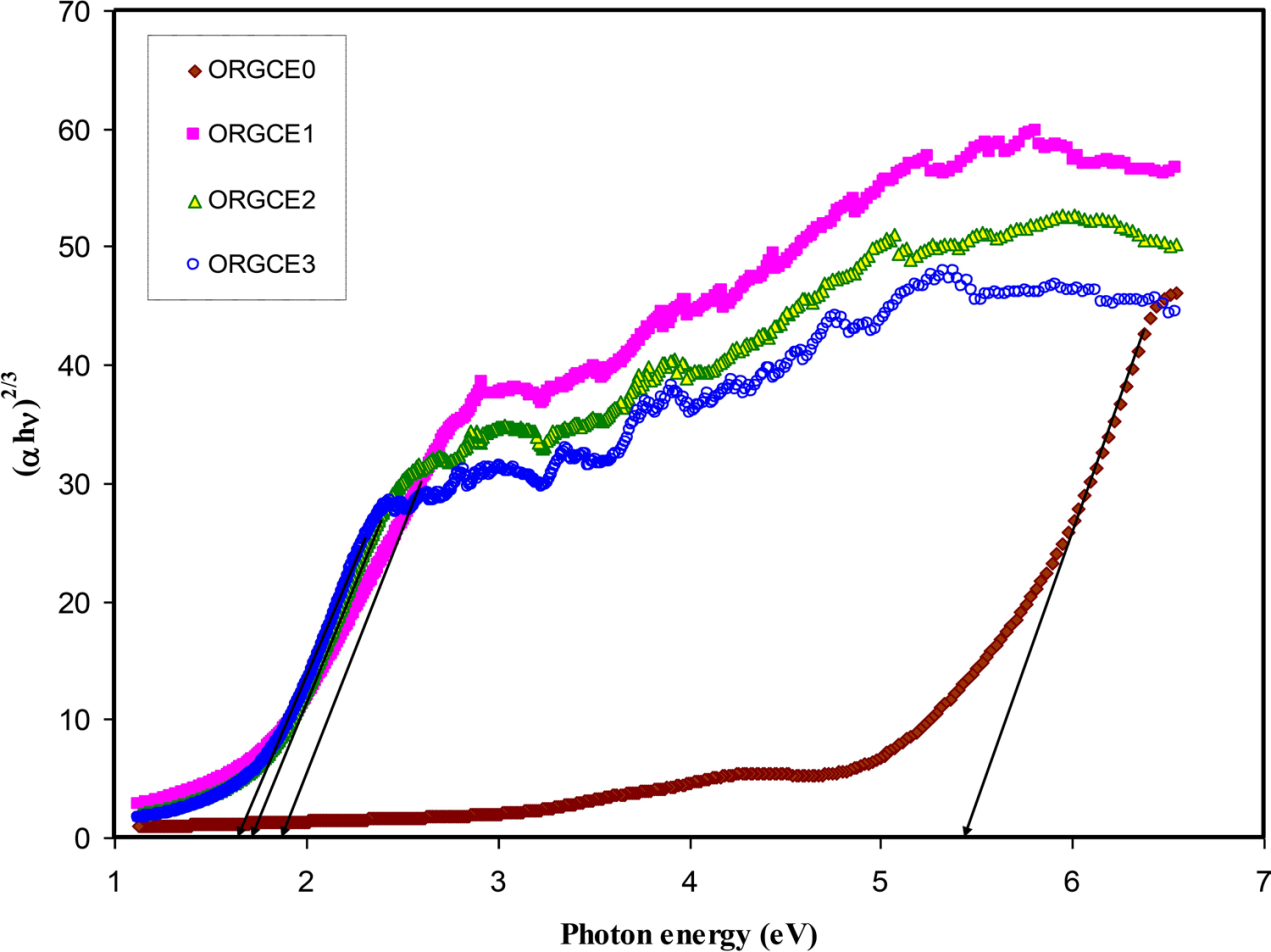
Plot of (αhυ)^2/3^ vs photon energy for pristine PVA (ORGCE0) and PVA composite films.

**Figure 14:**
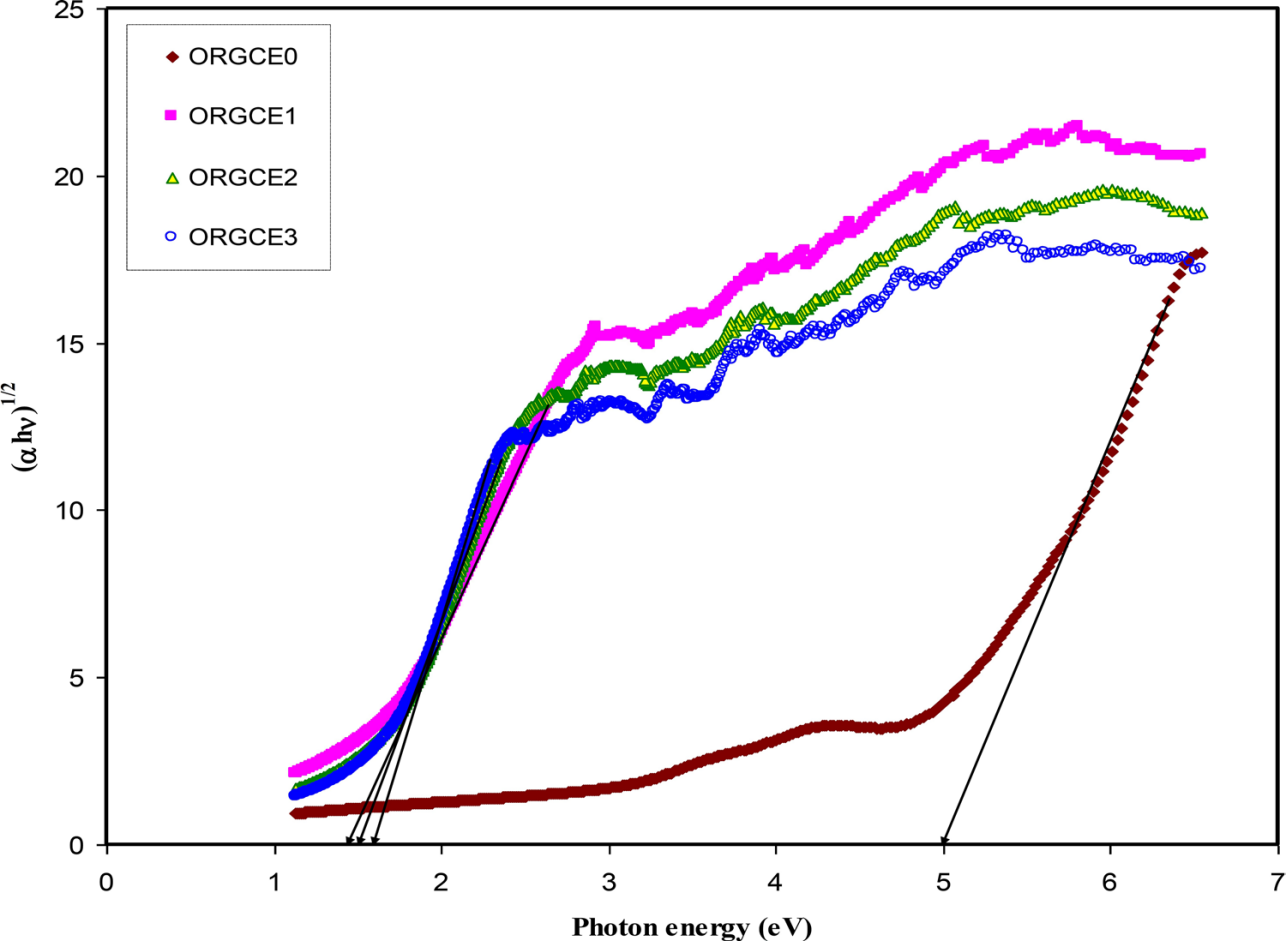
Plot of (αhυ)^1/2^ vs photon energy for pristine PVA (ORGCE0) and PVA composite films.

**Figure 15:**
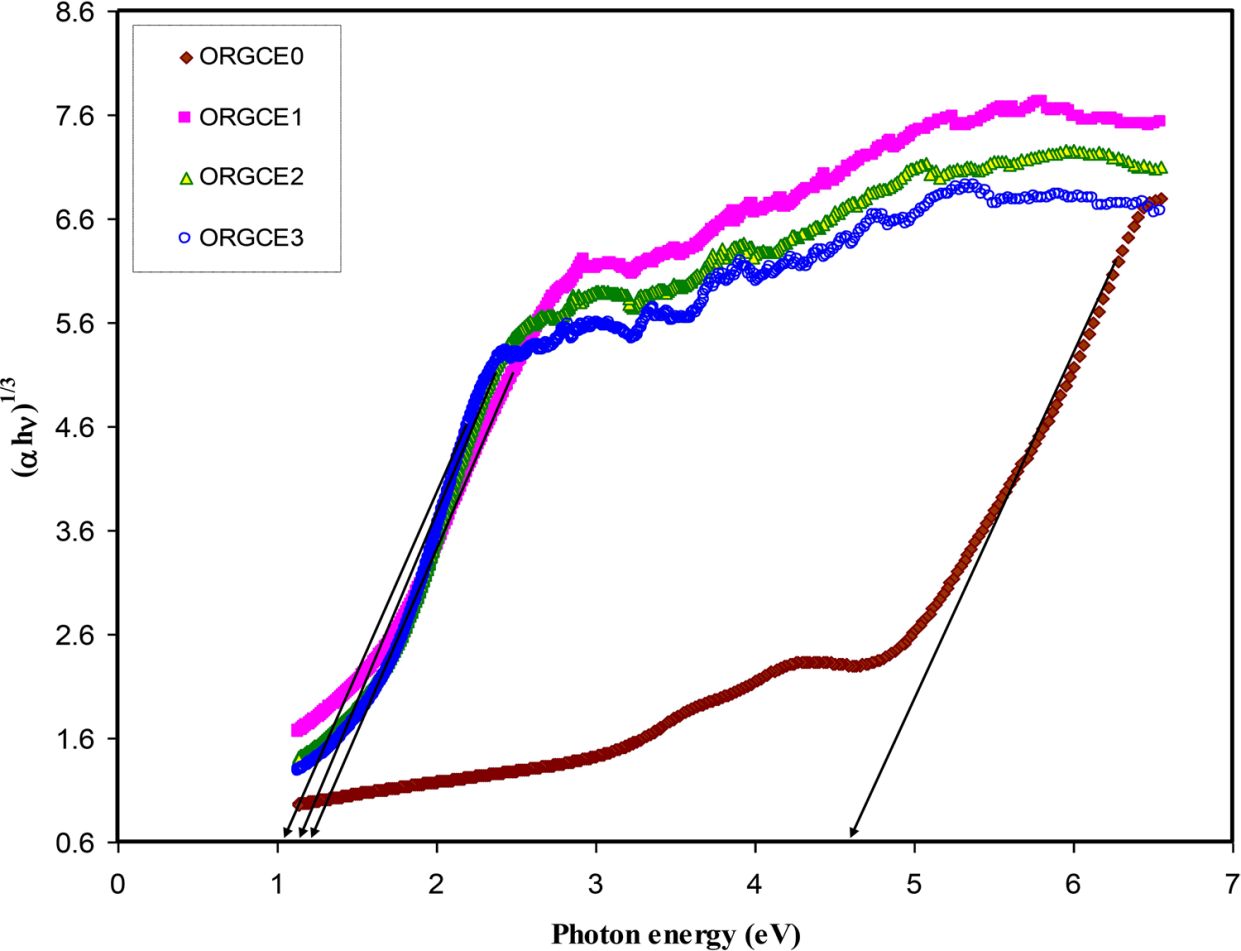
Plot of (αhυ)^1/3^ vs photon energy for pristine PVA (ORGCE0) and PVA composite films.

It was found that in the materials with having an amorphous structure, the band edges possess involvements by the various kinds of orbital of the metallic complex as well as the ligand; hence, it is not easy to forecast if the band is indirect or direct^82^. This study utilized ɛ_i_ parameter effectively to accurately determine the optical band gaps. It was determined quantum mechanically (microscopically) that the primary peak in the ɛ_i_ is related to the powerful interband transitions^74,80^. It is shown by the quantum mechanics perspective that there is a powerful relationship between the ɛ_i_ and the filled and unfilled electronic levels inside a solid^80^. The energy gap values obtained from the ɛ_i_ plot (Fig. 11) are compared with those values acquired from the Tauc's equation model (Figs. 12, 13, 14, 15) so as to determine the kinds of electronic transitions.

The fundamental absorption signifies band to band transitions that are made to follow particular selection rules. There are various kinds of transitions, which consistent with the band structure of a material^83,84^. The band gap energy refers to the magnitude of optical energy that needs to be gained by the electron to surpass the gap between the VB and the CB^25^. The band gap of the material can be identified by using the fundamental absorption that is related to the transition from the VB to CB.

In insulators/semiconductors, there are two kinds of band gaps, i.e. direct band gap and indirect band gap. Direct band gap is the band gap in which the value of the k-vector (momentum) of the lowest energy state in the CB and the highest energy state in the VB is the same. On the contrary, when the value is different, the band gap is indirect. In general, Tauc's model is usually employed to identify the optical band gap ( *E*_*g*_) in an amorphous semiconductor^66^. Potential transitions can be identified by plotting $({\mathrm{\alpha h\upsilon })}^{\frac{1}{\upbeta }}$ vs *hυ* to obtain the respective band gap by extrapolating the straight line part of the plot over the $h\upsilon$ axis to α = 0. **β** is ½ for direct allowed electron transitions, **β** is 2 for indirect allowed electron transitions, **β** is 3/2 for direct forbidden electron transitions, and **β** is 3 for indirect forbidden electron transitions^40^. The plot of $(\alpha h\upsilon )^{{\frac{1}{\beta }}}$ vs *hυ* for different values of the exponent has been shown in Figs. 12, 13, 14, 15, in accordance with the Tauc's model. When Figs. 12, 13, 14, 15 are compared with Fig. 11, it becomes possible to determine that the kind of electronic transition in pristine PVA and composites are direct allowed transition (**β** = 1/2) and direct forbidden transition (**β** = 3/2), respectively. Hence, it may be accepted that the optical dielectric function is a suitable technique for examining the solid band structure. Table 1 presents the band gap values acquired from the ɛ_i_ and the Tauc's model. It was affirmed by Yu et al.^80^ that the fundamental absorption edge acquired from the ɛ_i_ versus the photon energy should be the same or quite close to that acquired using the Tauc’s correlation.

**Table 1:** Measured optical band gap using Tauc's model and ɛ_i_ plot.

Sample code	E_g_ for β = 2	E_g_ for β = 1/2	E_g_ for β = 3	E_g_ for β = 3/2	E_g_ from ε_i_ plot
ORGCE0	5	6	4.8	5.45	5.8
ORGCE1	1.62	2.3	1.27	1.83	1.9
ORGCE2	1.55	2.1	1.2	1.79	1.8
ORGCE3	1.5	2	1	1.67	1.7

The findings of the current study show that it is critical to integrate metal-complexes with polar polymers like PVA so as to fabricate polymer composites with low energy band gaps. The optical band gap energy also lessens because of the rise in the degree of disorder within samples that emerges because of the modification in polymer structure^85,86^. The optical properties of PVA doped with different quantities of CeCl_3_ salt was examined by Abdelaziz^86^. The author found that there was a slight reduction in optical band gap of PVA following the inclusion of CeCl_3_ salt. The optical band gaps of the current work is quite low compared to those attained in the study of Abdelaziz and thus can be taken as a novel approach for developing polymer composites that have low optical band gap. It has been determined earlier that it is important to integrate inorganic compounds with organic polymers to develop organic–inorganic hybrid solar cells, which is fully taken to be a replacement for organic solar cells. This kind of solar cell has been shown by integrating organic semiconductors with various inorganic semiconductors to form the active layer of devices^87^. It was asserted by A.J. Heeger, the Noble prize winner, that: a critical part is played by the optical properties of a conducting polymer in comprehending the fundamental electronic structure of the material. He deduced that charge transfer complex in polymeric materials can be identified from their electronic spectra; therefore, spectroscopy is a robust method for characterization of the electronic processes that take place in the polymer within the undoped and doped states, and also while doping is being carried out^68^.

## Conclusions

In conclusion black tea extract solution were successfully used to synthesis Ce(III)-complex. This technique may be considered as a novel green method in the field of coordination chemistry for synthesis of different metal-complexes with low coast and high stability. The results of optical properties revealed that the combination of the Ce(III)-complex with PVA polymer is a unique method to fabricate polymer composites with small optical band gap energy. The FTIR, XRD, and UV–Vis results indicated that the reaction between cerium ions and polyphenols formed Ce(III)-complex. It is suggested by the XRD findings that synthesized Ce(III)-complexes were amorphous structure. It was shown by the FTIR spectra of the PVA/Ce(III)-complex composite films that there was a strong interaction between the functional groups of the pristine PVA and the Ce(III)-complex by shifting and decreasing the FTIR bands within the composite films in comparison to pristine PVA. The XRD deconvolution results also indicated that the amorphous phase in composite films increased. It is revealed in the AFM that the surface roughness in composite films raised with the rising of Ce(III)-complex. The complex issue of recognizing the nature of electron transition in condensed matter physics is overcome by examining the Tauc's model and optical dielectric loss parameter (ɛ_i_). Sufficient information was obtained from the point of view of quantum mechanics to confirm that a key role is played by ɛ_i_ parameter when analyzing the optical band gap.
